# CANDI: A Web Server for Predicting Molecular Targets and Pathways of Cannabis-Based Therapeutics

**DOI:** 10.21203/rs.3.rs-4744915/v1

**Published:** 2024-08-09

**Authors:** Srinivasan Ekambaram, Jian Wang, Nikolay V. Dokholyan

**Affiliations:** Penn State College of Medicine; Penn State College of Medicine; Penn State College of Medicine

**Keywords:** Cannabis, CANDI, Protein-Targets and Pathways

## Abstract

**Background:**

*Cannabis sativa* with a rich history of traditional medicinal use, has garnered significant attention in contemporary research for its potential therapeutic applications in various human diseases, including pain, inflammation, cancer, and osteoarthritis. However, the specific molecular targets and mechanisms underlying the synergistic effects of its diverse phytochemical constituents remain elusive. Understanding these mechanisms is crucial for developing targeted, effective cannabis-based therapies.

**Methods:**

To investigate the molecular targets and pathways involved in the synergistic effects of cannabis compounds, we utilized DRIFT, a deep learning model that leverages attention-based neural networks to predict compound-target interactions. We considered both whole plant extracts and specific plant-based formulations. Predicted targets were then mapped to the Reactome pathway database to identify the biological processes affected. To facilitate the prediction of molecular targets and associated pathways for any user-specified cannabis formulation, we developed CANDI (Cannabis-derived compound Analysis and Network Discovery Interface), a web-based server. This platform offers a user-friendly interface for researchers and drug developers to explore the therapeutic potential of cannabis compounds.

**Results:**

Our analysis using DRIFT and CANDI successfully identified numerous molecular targets of cannabis compounds, many of which are involved in pathways relevant to pain, inflammation, cancer, and other diseases. The CANDI server enables researchers to predict the molecular targets and affected pathways for any specific cannabis formulation, providing valuable insights for developing targeted therapies.

**Conclusions:**

By combining computational approaches with knowledge of traditional cannabis use, we have developed the CANDI server, a tool that allows us to harness the therapeutic potential of cannabis compounds for the effective treatment of various disorders. By bridging traditional pharmaceutical development with cannabis-based medicine, we propose a novel approach for botanical-based treatment modalities.

## Introduction

*Cannabis sativa* is among the most ancient cultivated plants, with evidence suggesting its utilization may date back nearly a million years(Ren et al., 2021). Its multifaceted advantages, particularly as a source of fiber, have resulted in its extensive function in both agricultural and industrial applications(Fordjour et al., 2023; H.-L. Li, 1974). Currently, cannabis is consumed for medicinal and recreational purposes and is recognized for its various derived metabolites, including terpenoids, flavonoids, sterols, and phytocannabinoids(Simiyu et al., 2022). Phytocannabinoid compounds are being comprehensively reviewed and are stated to interrelate with a complex network of receptors and signaling pathways that play a crucial role in modulating various physiological processes, including pain perception, appetite, mood, and memory(Bonn-Miller et al., 2018; Pacher et al., 2006; Zou & Kumar, 2018). The principal psychoactive constituent of cannabis, Δ9-tetrahydrocannabinol (THC), has been the focus of wide-ranging investigation and is the only approved cannabinoid-based prescription for the healing of chemotherapy-induced sickness in patients(Badowski, 2017; Ng et al., 2024). However, the therapeutic potential of cannabis extends far beyond THC, with numerous other cannabinoids and terpenes exhibiting promising pharmacological activities(Alves et al., 2020).

On the other hand, cannabis, a formerly banned substance universally, has endured a substantial shift in perception, with various countries like the United States and Canada acknowledging its long-standing traditional medicinal use and legalizing its usage. This paradigm shift has been driven by scientific research and an emerging understanding of the potential therapeutic benefits of cannabis and its active compounds(Dalli et al., n.d.). Modern computational and experimental studies on phytocannabinoids and other cannabis-derived compounds have elucidated their medicinal value in the treatment of diverse human disorders, including inflammatory bowel disease (IBD), cancer, Alzheimer’s disease, Parkinson’s disease, and multiple sclerosis(Abd-Nikfarjam et al., 2023; Carkaci-Salli et al., 2023; Cassano et al., 2020; Fadaka et al., 2022; Farrelly et al., 2021; Helcman & Šmejkal, 2022; Hryhorowicz et al., 2021; Varshney et al., 2023). Consequently, the integration of cannabis-based therapeutics into conventional medical practice continues to expand, offering new treatment avenues and improved outcomes for patients with these debilitating conditions(Scherma et al., 2020). Hence, the historical significance and value of cannabis have further emphasized the importance of cannabis-based drug discovery, driving advancements in our understanding of its therapeutic potential and facilitating its integration into modern medical practice.

Naturally occurring chemical compounds from various sources are vital in diverse biological activities and are at the forefront of drug discovery studies. However, identifying the targets for these compounds remains a bottleneck in understanding their mechanisms of action(G. Li et al., 2021; Newman & Cragg, 2016). Experimental techniques, such as affinity chromatography, protein microarrays, and genomic or proteomic studies, are typically employed for target identification, but they are highly time-consuming and relatively expensive(Cheng et al., 2011; Zhang et al., 2022). In contrast to the traditional drug development strategies, it is widely known that compounds often interact with multiple targets, presenting a potential limitation for the experimental approaches(G. Li et al., 2021). Computational methods offer an alternative by employing various algorithms to identify targets for compounds. For instance, models, such as network-based approaches, data mining, and machine learning, have been used to predict targets for compounds(Agamah et al., 2019; Ezzat et al., 2019; Nogueira & Koch, 2019). Moreover, the recent development of deep learning networks has expanded the scope and improved the predictability of target identification from various biological databases that have grown enormously with abundant data on protein-ligand complexes. Deep learning models can effectively analyze large datasets and complex biological networks, making them increasingly valuable in modern drug target identification(Askr et al., 2023; Chen et al., 2024; Zeng et al., n.d.; Zhou et al., 2023). DRIFT is one such model that helps map the targets for the compounds using deep learning approaches by integrating neural network architecture to predict the target-compound binding affinity using the Yuel algorithm in the backend(Chirasani et al., 2022; Wang & Dokholyan, 2022). Hence, advancing computational methodologies for discerning compound-target correlations and extrapolating potential targets for pharmaceuticals and bioactive substances through amalgamating and integrating critical target data from myriad sources provides a valid approach to understanding the context of compound-target interactions. Furthermore, pathways play a pivotal role in elucidating the intricate nature of various diseases, as proteins function within complex networks of interactions(Liu & Chance, 2013). Complex diseases often arise from the dysregulation of multiple targets within interconnected pathways or variations in different genes within the same pathways across diverse patient populations(Y.-A. Kim et al., 2011). Hence, elucidating the relationship between targets and disease-associated pathways is crucial for comprehending disease mechanisms and holds promise for developing efficacious treatments.

Despite significant advances, several critical knowledge gaps persist in our understanding of cannabis pharmacology and its therapeutic potential. There remains a need for further exploration into their mechanisms of action, efficacy, and safety profiles. Furthermore, the variability in cannabis strains, lack of standardized formulations, and potential adverse effects associated with long-term use pose significant challenges to the development of cannabis-based therapeutics. Addressing these gaps is imperative for advancing our understanding of cannabis pharmacology and translating it into safe and effective treatments for a wide range of disorders. In light of these considerations, we have focused the study on cannabis-based drug discovery, which aims to harness the synergistic effects of the plant’s diverse phytochemical constituents, a phenomenon known as the “entourage effect.” This strategy recognizes that the therapeutic efficacy of cannabis may not be solely attributable to a single compound but rather to the intricate interplay between multiple cannabinoids, terpenes, and other compounds present in the plant(Ferber et al., 2020; Koltai & Namdar, 2020). Accruing data from numerous studies suggests that cannabis extracts or combinations of cannabis-derived compounds may elicit synergistic effects in alleviating pain, reducing inflammation, and mitigating the psychoactive effects(Anand et al., 2021; Bonn-Miller et al., 2018a; Chacon et al., 2022; Namdar et al., 2020; Sepulveda et al., 2022). Hence, utilizing computational algorithms, we aim to shed light on the intricate interplay between cannabinoids, terpenes, and other compounds, with the ultimate goal of contributing to the development of novel and efficacious cannabis-based therapeutics. Therefore, leveraging computational algorithms, we seek to elucidate the complex synergistic interactions between cannabinoids, terpenes, and other bioactive constituents within the cannabis plant. This multi-faceted approach aims to identify potential therapeutic targets, optimize drug formulations, and ultimately contribute to the development of innovative and effective cannabis-based therapies for a wide range of medical conditions. Furthermore, we have developed a user-friendly web interface (CANDI, http://candi.dokhlab.org) to facilitate the prediction of targets and relevant pathways for cannabis compounds and formulations, thereby streamlining the drug discovery process and enhancing accessibility for researchers and clinicians alike. Hence, our study contributes to the advancement of drug discovery efforts aimed at harnessing the therapeutic potential of cannabis compounds for the effective treatment of various disorders.

## Materials and Methods

### Data Curation and Compilation.

The initial dataset comprising compounds sourced from the cannabis plant was curated from Pennsylvania state-approved keystone state testing – cannabis laboratory(Raup-Konsavage et al., 2020). These compounds were systematically classified into three main categories: cannabinoids, terpenes, and flavonoids. In total, 73 compounds, with 16 falling under cannabinoids, 39 under terpenes, and 18 under flavonoids, as detailed in Table 1.

### Target prediction.

Targets for the cannabis compounds were initially determined using the DRIFT algorithm, with the SMILES notation serving as the input format. Subsequently, the obtained targets were refined to include only protein-related factors. The resultant sorted targets and their respective scores were then utilized for subsequent analyses. Further, the results were structured in a matrix (C,T), where C represents compounds and T represents targets, each with corresponding predicted scores. Subsequently, for each compound, the user-provided formulation was incorporated as weights $\left(W_{j}\right)$, which were then multiplied with the corresponding scores in the matrix (MScore). The resulting values were summed over all targets (j=1ton), yielding a final score for each compound.


$$Target\;scores\;=\sum_{j=1}^{n}MScore\;*\;W_{j}$$

This computation yielded the finalized results, presented in concatenated form, which were subsequently sorted according to normalization criteria. Ultimately, the targets associated with the user-provided formulation for the set of compounds were obtained, along with their normalized scores.

### Pathway Mapping.

We undertook a systematic curation process to map the pathways associated with the identified targets utilizing data from the REACTOME database(Milacic et al., 2024). Initially, the mapping of UniProt identifiers to pathways was facilitated through an in-house Python script. Subsequently, the UniProt identifiers and their corresponding normalized scores derived from the target analysis were employed as input for pathway prediction. Notably, these scores were utilized as weights during the prediction process. The mapping procedure involved querying the REACTOME database to retrieve pathways associated with the identified UniProt entries. The retrieved pathways were concatenated, forming a comprehensive list. To rank the pathways, we utilized pathway scores. To compute the pathway scores, the weights of the UniProt identifiers mapped to each pathway were aggregated and divided by the total number of UniProt identifiers provided as input.

$$Pathway\;score\;=\sum (MW)\;*\;\frac{NM}{NT}$$

where MW, NM, and NT represent mapped target weights, the number of mapped targets, and the number of total targets, correspondingly.

This systematic approach ensured the accurate prediction of pathways associated with the identified targets, enhancing our understanding of the biological processes influenced by the investigated compounds.

### Compound-Target-Pathway Similarity Analysis.

To assess the relationship between the compounds and targets, we have utilized the DRIFT predictions on the cannabis compounds to establish an indirect relationship between them. We leveraged the target information and scores to generate vector representations for each compound. These vectors served as the basis for computing cosine similarity scores, enabling the quantification of compound-target relationships.

$$\cos\theta =\frac{A\cdot B}{∥A∥∥B∥},$$

where A and B represent the vectors corresponding to two compounds. The computed similarity scores were visualized as a heatmap using the Matplotlib library in Python. This visualization method provided users with an intuitive means to comprehend the degree of similarity between compounds and their associated targets. The approach allowed for a clear representation of the complex relationships within the dataset, enabling researchers to rapidly identify patterns and potential areas of interest. We extended our analysis by curating pathways associated with the compounds using the Reactome database, a comprehensive open-source database of human biological processes. This additional step allowed us to map the similarity between compounds and their related pathways, providing a more holistic view of the potential biological impacts of these substances.

### Construction of CANDI Web Interface.

We developed a user-friendly web interface using Flask, HTML5, CSS, and JavaScript. HTML5 was utilized to structure the content of the web pages, while CSS3 was employed for styling and layout customization. JavaScript was integrated to enhance user interactivity and functionality, ensuring a seamless browsing experience. Python Flask was used to handle data retrieval and processing tasks for back-end development. The compatibility of CANDI was tested across popular web browsers such as Chrome and Firefox to ensure consistent performance and rendering. Overall, CANDI provides users with an intuitive and versatile platform for accessing and analyzing cannabis compound data.

### CANDI Web Interface.

**Figure 1:**
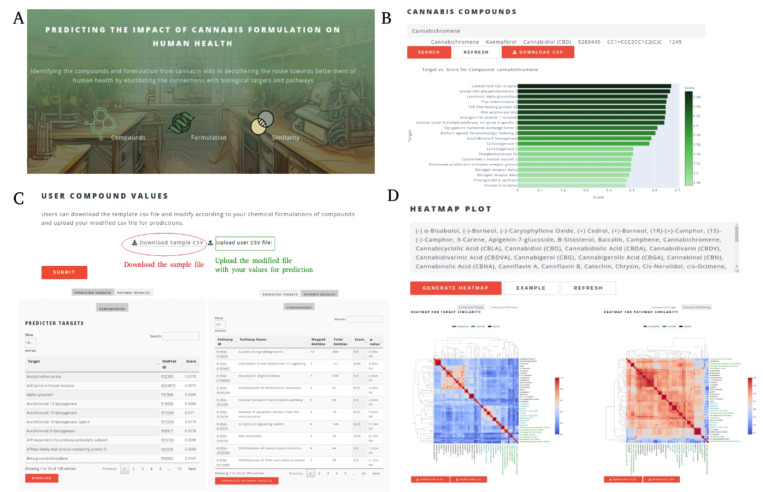
The overall functioning of the CANDI webserver. (A) Front page of the CANDI user interface for the compounds, formulation and similarity functions. (B) The compound information data could be obtained for all 73 cannabis compounds using common names, SMILES, and PubChem IDs. By browsing the compound information, users can intuitively obtain targets for the compound with a predicted score, pharmacophore-similar compounds with similarity values, assay value and type, and graphical representation of the targets vs. score as a bar plot. (C) The Formulation page lets users download the file to add user values and upload the file for the target and pathway prediction. (i) Predicted targets are ranked according to the score and linked to their corresponding uniport entries. (ii) Pathways were mapped for the predicted targets from the Reactome database, and the pathway score was shown as an interactive table. (D) The compound-target-pathway similarly page allows the user to provide input for the cannabis compounds to identify the relationship between the compound-target and compound-pathway. The output is a heatmap with the download option for the image file and data in CSV format.

CANDI offers a suite of interactive modules, each tailored to address distinct stages of cannabis-based drug discovery (Fig. 1A)

#### Compound Search:

This module serves as a comprehensive repository of information on individual cannabis-derived compounds (Fig. 1B). Users may search for specific compounds using various formats, including generic names, SMILES strings, and PubChem IDs. Upon searching, users can access detailed data, including the function to download the results in table format.

#### Predicted Molecular Targets:

A curated list of proteins or receptors likely to interact with the compound is provided based on experimental evidence and computational predictions with corresponding predicted scores. The interface also includes a bar plot to represent the targets and their scores visually.

#### Similarity Search Results:

A list of structurally similar compounds and similarity scores calculated using the FP2 fingerprint and SMILES strings are provided to explore potential analogs with enhanced or altered pharmacological profiles.

#### Assay Data:

When available, results from relevant biological assays are presented, offering insights into the compound’s potency with a value alongside the assay method.

#### Formulation:

Recognizing the importance of the entourage effect, this module allows users to input a specific formulation of multiple cannabis compounds. CANDI then leverages its underlying algorithms to efficiently predict the target and map its relevant pathways (Fig. 1C). Potential molecular targets that the specific combination of compounds in the formulation may uniquely or preferentially modulate are identified. The associated biological pathways likely to be impacted by the formulation are mapped highlighting potential therapeutic applications.

#### Compound-target-pathway Similarity:

This module facilitates target-based drug discovery by enabling users to identify novel cannabis compounds based on their relationship to specific targets and pathways (Fig. 1D). Users can input a set of cannabis compounds, and CANDI employs a cosine similarity algorithm to assess the similarity between the input compounds, known targets and pathways. This analysis identifies cannabis compounds predicted to interact with similar and potentially distinct targets and pathways. This method enhances our understanding of individual compounds and illuminates the complex network of interactions within biological systems. By providing a holistic view of the relationships between compounds and their targets, this feature aids in discerning combinations of compounds that may synergistically modulate multiple targets within a given pathway.

## Results and Discussion

*Cannabis sativa* exhibits promising therapeutic potential, substantiated by accumulating scientific evidence. However, the development of standardized cannabis-based therapeutics is hampered by challenges inherent to the plant’s phytochemical complexity. We employ a deep learning computational approach to predict molecular targets and associated pathways for cannabis formulations, elucidating the synergistic effect. This research is facilitated by CANDI, a user-friendly web server designed to analyze compound-target interactions and therapeutic mechanisms comprehensively.

### Architecture of CANDI.

**Figure 2:**
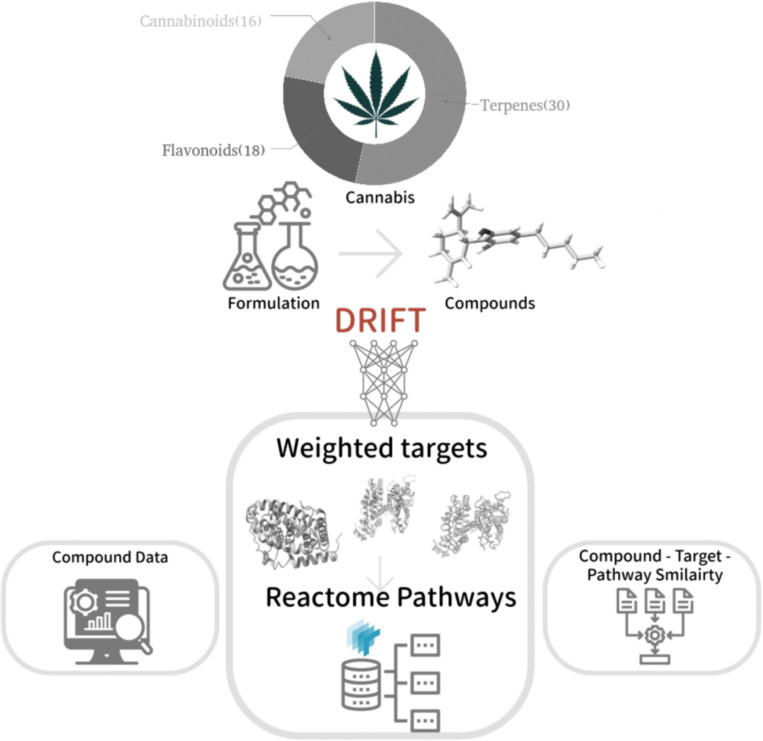
The workflow adapted in this study to identify the targets and map associated pathways for the formulation of cannabis compounds.

The CANDI web server is an integrated computational platform designed to facilitate the identification of molecular targets and associated pathways for user-specified formulations of cannabis-derived compounds (Fig. 2). The workflow is instigated by user input, wherein the specific combination and concentrations of cannabinoids, terpenes, and other relevant molecules of interest are defined. Leveraging the DRIFT algorithm(Chirasani et al., 2022), a deep learning model trained on structural and chemical properties, the platform predicts potential targets for the compounds. It assigns scores based on the likelihood of interaction. These scores are normalized and re-ranked, considering the user-specified formulation composition and concentrations. The ranked targets are mapped to their corresponding UniProt identifiers(The UniProt Consortium, 2023), enabling the identification of relevant pathways within the Reactome database(Milacic et al., 2024), a comprehensive resource of biological pathways and processes. The final output provided by CANDI is a ranked list of pathways, weighted by the number and scores of associated targets, offering insights into the potential mechanisms underlying the therapeutic effects of the specified cannabis formulation. This integrated computational approach enables researchers to systematically explore the intricate interplay between cannabis compounds and their molecular targets, accelerating the development of targeted therapies and elucidating the mechanistic underpinnings of cannabis-based therapeutics.

### Case Study on Cannabis Oil Formulation.

To validate CANDI’s functionality, performed studies utilizing a commercial cannabis oil formulation comprising various composition of cannabinoids and terpenes (Table 2). The formulation’s composition, obtained from experimental data, was input into CANDI by modifying the platform’s sample CSV file. Upon analysis, CANDI generated results that were presented in two sections: predicted molecular targets and associated pathways.

**Figure 3:**
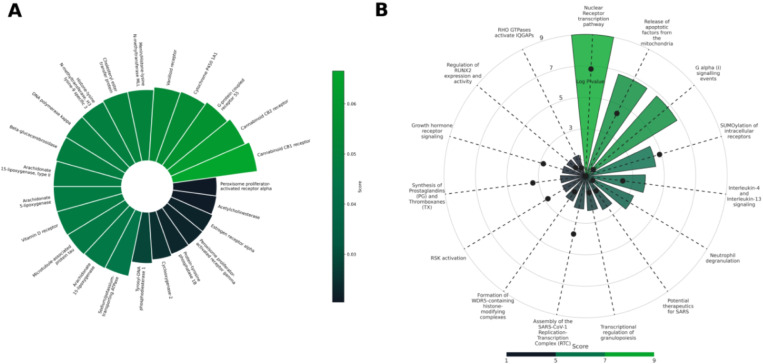
A case study on cannabis oil from the experimental studies. (A) The predicted targets for the cannabis oil formulation were ranked according to the predictive scores suggesting that CB1 and CB2 are top targets for the given formulation (B) Mapped pathways for the targets predicted were plotted elucidating the involvement of the targets in nuclear receptor transcription factor and G alpha (i) signaling events pathways.

The predicted targets section displayed a ranked list, ordered by their predicted interaction scores, with the highest-scoring targets listed first. For further reference, each target was linked to its corresponding UniProt entry. The analysis revealed that the formulation was predicted to interact with cannabinoid receptors CB1 and CB2, followed by G protein-coupled receptor 55 (GPR55), cytochrome P450 enzymes, and other receptors (Fig. 3A). The associated pathways section provided a detailed overview of the Reactome pathways linked to the predicted targets. These pathways were ranked based on their predictive score. Among the identified pathways were nuclear receptor transcription, G alpha(i) signaling events, the release of apoptotic factors from mitochondria, and SUMOylation of intracellular receptors all implicated in various physiological processes (Fig. 3B). Hence, the analysis revealed that this formulation could modulate multiple targets and pathways associated with pain management, inflammation, and neurological disorders. The formulation was predicted to interact with the endocannabinoid system, including the CB1 and CB2 receptors. These interactions could contribute to the formulation’s potential analgesic, anti-inflammatory, and neuroprotective effects(Donvito et al., 2018; Gonzalo-Consuegra et al., 2024). Furthermore, the analysis identified several relevant pathways related to pain perception and inflammation (Che, 2021; Zhao et al., 2020). Hence, CANDI-generated hypothesis is that this formulation shows promise as a potential therapeutic agent for these conditions. Further research, including preclinical and clinical studies, is warranted to validate these findings and explore the full therapeutic potential of this formulation.

### Case Study on Cannabinoids.

In the second case study, we analyzed a cannabis oil formulation containing only cannabinoids. From the analyses we could decipher that the formulation was predicted to interact with cannabinoid receptors CB1 and CB2, DNA polymerase kappa, and G protein-coupled receptor 55 (GPR55), vitamin receptor, and other receptors (Supp. Figure 1A). The associated pathways were G alpha(i) signaling events, Interlukin-4 and Interlukin-13 signaling, and neutrophil degranulation, entirely associated in several biological activities (Supp. Figure 2B). Notably, this formulation was predicted to interact with the well-characterized CB1 and CB2 receptors, which are primary targets in cannabinoid research. These receptors are involved in various physiological processes, including pain modulation and inflammatory responses(Raup-Konsavage et al., 2023; Turcotte et al., 2016). In accord with our findings, the mapped pathways, particularly G alpha(i) signaling events and Interleukin-4 and Interleukin-13 signaling, have been implicated in pain perception and inflammatory processes(Ibsen et al., 2017; Oláh et al., 2017).

### Case Study on Terpenes.

In the third case study, a formulation containing only terpenes was analyzed. Predicted targets and associated pathways were charted. The formulation was predicted to interact with solute carrier organic anion transporter family members 1B1 and 1B3, bile acid receptor FXR, arachidonate 15-lipoxygenase receptors and also cannabinoid CB2 receptor (Supp. Figure 2A). Associated pathways included nuclear receptor transcription, aspirin ADME, SUMOylation of intracellular receptors, Interleukin-4 and Interleukin-13 signaling, and G alpha (i) signaling events all implicated in various biological processes (Supp. Figure 2B). These findings suggest that this terpene formulation may contribute to modulating diverse physiological functions through its interactions with these targets and pathways. In accord with our findings, the identified targets and pathways are commonly involved in inflammatory bowel disorders, various inflammatory diseases, and metabolic disorders(Del Prado-Audelo et al., 2021; T. Kim et al., 2020; LaVigne et al., 2021).

## Conclusion

The development of cannabis-based therapeutics holds significant potential for treating diverse medical conditions. However, this potential is constrained by the intricacy of the cannabis plant and the current lack of standardized, targeted therapies. The study exemplifies a noteworthy improvement in overcoming these challenges by leveraging computational approaches, specifically deep learning algorithms. CANDI facilitates the identification of molecular targets and associated pathways for specific combinations of cannabis-derived compounds, addressing research gaps related to the entourage effect. Additionally, the user-friendly interface allows researchers to investigate the complex interplay between these compounds and their potential therapeutic targets. By integrating information on compound-target interactions and relevant biological pathways, CANDI facilitates a comprehensive analysis of the molecular mechanisms underlying the therapeutic effects of cannabis formulations and offers a plausible hypothesis on health outcomes of such compounds and formulations. Hence, the study contributes to the advancement of drug discovery efforts aimed at harnessing the therapeutic potential of cannabis compounds for the effective treatment of various disorders.

## Figures and Tables

**Table 1:** A curated dataset of 73 compounds extracted from *Cannabis sativa* through experimental studies. This dataset encompassed 16 cannabinoids, 39 terpenes, and 18 flavonoids.

S.no	Cannabis Compounds
Cannabinoids	
1	Cannabichromene
2	Cannabichromenic Acid
3	Cannabidiol (CBD)
4	Cannabidiolic Acid (CBDA)
5	Cannabidivarin (CBDV)
6	Cannabidivarinic Acid (CBDVA)
7	Cannabigerol (CBG)
8	Cannabigerolic Acid (CBGA)
9	Cannabicyclolic Acid (CBLA)
10	Cannabinol (CBN)
11	Cannabinolic Acid (CBNA)
12	Delta8-Tetrahydrocannabinol (d8-THC)
13	Tetrahydrocannabinolic Acid (THCA)
14	Tetrahydrocannabivarin (THCV)
15	Tetrahydrocannabivarinic Acid (THCVA)
16	Delta9-Tetrahydrocannabinol (d9-THC)
Terpenes	
17	β-Farnesene
18	β-Caryophyllene
19	α-Humulene
20	α-Farnesene
21	(−) α-Bisabolol
22	β-Myrcene
23	R (+) Limonene
24	3-Carene
25	Endo-Fenchyl Alcohol
26	α-Terpineol
27	Guaiol
28	α-Pinene
29	Linalool
30	(−)-Caryophyllene Oxide
31	Camphene
32	α-Terpinene
33	Eucalyptol
34	ɣ-Terpinene
35	Fenchone
36	Trans-Nerolidol
37	(1R)-(+)-Camphor
38	Valencene
39	(+) Cedrol
40	Cis-Nerolidol
41	L-Fenchone
42	α-Phellandrene
43	Hexahydro Thymol
44	α-Cedrene
45	Geranyl Acetate
46	cis-Ocimene
47	(−)-Borneol
48	β-pinene
49	Terpinolene
50	Nerol
51	Trans-Ocimene
52	Sabinene
53	(1S)-(−)-Camphor
54	Isoborneol
55	(+)-Borneol
Flavonoids	
56	Kaempferol
57	Luteolin
58	Vitexin
59	Rutin
60	Chrysin
61	Baicalin
62	Orientin
63	Quercetin-3-glucoside
64	Isovexitin
65	Luteolin-7-o-glucoside
66	Apigenin-7-glucoside
67	Catechin
68	Quercetin
69	Epicatechin
70	Epigalocatechin
71	Cannflavin B
72	B-Sitosterol
73	Cannflavin A

**Table 2:** Dataset of the cannabis compounds with formulation used for the case study

Compounds	Cannabis oil	Cannabinoids	Terpenes
Cannabichromene	1.55	3.89	0
Cannabidiol (CBD)	36.56	34.87	0
Cannabidivarin (CBDV)	0	0	0
Cannabidivarinic Acid (CBDVA)	0	61.09	0
Cannabigerol (CBG)	35.63	21.31	0
Cannabigerolic Acid (CBGA)	0	8.45	0
Cannabicyclolic Acid (CBLA)	0	0.13	0
Cannabinol (CBN)	0	0.15	0
Tetrahydrocannabivarin (THCV)	0	0.19	0
Tetrahydrocannabivarinic Acid (THCVA)	0	0.34	0
Delta8-Tetrahydrocannabinol (d8-THC)	0	0	0
Delta9-Tetrahydrocannabinol (d9-THC)	0	2.64	0
(−)-Caryophyllene Oxide	0	0	0.47
(+) Cedrol	0	0	0.09
(−) α-Bisabolol	0.04	0	1.58
α-Cedrene	0	0	0.07
α-Farnesene	0	0	1.49
α-Humulene	0.023	0	1.32
α-Phellandrene	0	0	0.04
α-Pinene	0	0	0.01
α-Terpineol	0.001	0	0.09
β-Farnesene	0	0	5.46
β-Myrcene	0.001	0	0.25
β-pinene	0	0	0.02
Camphene	0	0	0.01
Cis-Nerolidol	0	0	0.02
cis-Ocimene	0	0	0.03
Endo-Fenchyl Alcohol	0.002	0	0.08
Eucalyptol	0	0	0.01
Fenchone	0	0	0.01
ɣ-Terpinene	0	0	0.01
Geranyl Acetate	0	0	0.01
Guaiol	0.04	0	0.56
Linalool	0	0	0.07
R (+) Limonene	0.003	0	0.12
Terpinolene	0	0	0.25
β-Caryophyllene	0.091	0	5.37
Trans-Nerolidol	0	0	0.11
Valencene	0	0	0.72

## Data Availability

All data generated or analysed during this study are included in this published article and its supplementary information.

## References

[R1] Abd-Nikfarjam B, Dolati-Somarin A, Baradaran Rahimi V, Cannabinoids in neuroinflammatory disorders: Focusing on multiple sclerosis, Parkinsons, and Alzheimers diseases. BioFactors 2023;49(3):560–583. 10.1002/biof.1936. 36637897

[R2] Agamah FE, Mazandu GK, Hassan R, Computational/in silico methods in drug target and lead prediction. Brief Bioinform 2019;21(5):1663–1675. 10.1093/bib/bbz103. PMC7673338 31711157

[R3] Alves P, Amaral C, Teixeira N, Cannabis sativa: Much more beyond Δ9-tetrahydrocannabinol. Pharmacological Research 2020;157:104822. 10.1016/j.phrs.2020.104822. 32335286

[R4] Anand U, Pacchetti B, Anand P, Cannabis-Based Medicines and Pain: A Review of Potential Synergistic and Entourage Effects. Pain Management 2021;11(4):395–403. 10.2217/pmt-2020-0110. 33703917

[R5] Askr H, Elgeldawi E, Aboul Ella H, Deep learning in drug discovery: an integrative review and future challenges. Artif Intell Rev 2023;56(7):5975–6037. 10.1007/s10462-022-10306-1. 36415536 PMC9669545

[R6] Badowski ME. A review of oral cannabinoids and medical marijuana for the treatment of chemotherapy-induced nausea and vomiting: a focus on pharmacokinetic variability and pharmacodynamics. Cancer Chemother Pharmacol 2017;80(3):441–449. 10.1007/s00280-017-3387-5. 28780725 PMC5573753

[R7] Bonn-Miller MO, ElSohly M, Loflin MJE, Cannabis and Cannabinoid Drug Development: Evaluating Botanical Versus Single Molecule Approaches. Int Rev Psychiatry 2018;30(3):277–284. 10.1080/09540261.2018.1474730. 30179534 PMC6242809

[R8] Bonn-Miller MO, ElSohly M, Loflin MJE, Cannabis and Cannabinoid Drug Development: Evaluating Botanical Versus Single Molecule Approaches. Int Rev Psychiatry 2018;30(3):277–284. 10.1080/09540261.2018.1474730. 30179534 PMC6242809

[R9] Carkaci-Salli N, Raup-Konsavage WM, Karelia D, Cannabinoids as Potential Cancer Therapeutics: The Concentration Conundrum. Cannabis and Cannabinoid Research 2023. 10.1089/can.2022.0344. 36944160

[R10] Cassano T, Villani R, Pace L, From Cannabis sativa to Cannabidiol: Promising Therapeutic Candidate for the Treatment of Neurodegenerative Diseases. Front Pharmacol 2020;11. 10.3389/fphar.2020.00124. PMC7069528 32210795

[R11] Chacon FT, Raup-Konsavage WM, Vrana KE, Secondary Terpenes in Cannabis sativa L.: Synthesis and Synergy. Biomedicines 2022;10(12):3142. 10.3390/biomedicines10123142. 36551898 PMC9775512

[R12] Che T. Advances in the Treatment of Chronic Pain by Targeting GPCRs. Biochemistry 2021;60(18):1401–1412. 10.1021/acs.biochem.0c00644. 33186495

[R13] Chen H, King FJ, Zhou B, Drug target prediction through deep learning functional representation of gene signatures. Nat Commun 2024;15(1):1853. 10.1038/s41467-024-46089-y. 38424040 PMC10904399

[R14] Cheng T, Li Q, Wang Y, Identifying Compound-Target Associations by Combining Bioactivity Profile Similarity Search and Public Databases Mining. J Chem Inf Model 2011;51(9):2440–2448. 10.1021/ci200192v. 21834535 PMC3180241

[R15] Chirasani VR, Wang J, Sha C, Whole proteome mapping of compound-protein interactions. Current Research in Chemical Biology 2022;2:100035. 10.1016/j.crchbi.2022.100035. 38125869 PMC10732549

[R16] DalliM, AziziS, AzgharA, Cannabis sativa L.: A comprehensive review on legislation, decriminalization, phytochemistry, antimicrobial activity, and safety. J Food Drug Anal n.d.;31(3):408–435. 10.38212/2224-6614.3471.39666278

[R17] Del Prado-Audelo ML, Cortés H, Caballero-Florán IH, Therapeutic Applications of Terpenes on Inflammatory Diseases. Front Pharmacol 2021;12:704197. 10.3389/fphar.2021.704197. 34483907 PMC8414653

[R18] Donvito G, Nass SR, Wilkerson JL, The Endogenous Cannabinoid System: A Budding Source of Targets for Treating Inflammatory and Neuropathic Pain. Neuropsychopharmacol 2018;43(1):52–79. 10.1038/npp.2017.204. PMC5719110 28857069

[R19] Ezzat A, Wu M, Li X-L, Computational prediction of drug-target interactions using chemogenomic approaches: an empirical survey. Brief Bioinform 2019;20(4):1337–1357. 10.1093/bib/bby002. 29377981

[R20] Fadaka AO, Taiwo OA, Dosumu OA, Computational prediction of potential drug-like compounds from Cannabis sativa leaf extracts targeted towards Alzheimer therapy. Journal of Molecular Liquids 2022;360:119393. 10.1016/j.molliq.2022.119393.

[R21] Farrelly AM, Vlachou S, Grintzalis K. Efficacy of Phytocannabinoids in Epilepsy Treatment: Novel Approaches and Recent Advances. International Journal of Environmental Research and Public Health 2021;18(8):3993. 10.3390/ijerph18083993. 33920188 PMC8070313

[R22] Ferber SG, Namdar D, Hen-Shoval D, The “Entourage Effect”: Terpenes Coupled with Cannabinoids for the Treatment of Mood Disorders and Anxiety Disorders. Curr Neuropharmacol 2020;18(2):87–96. 10.2174/1570159X17666190903103923. 31481004 PMC7324885

[R23] Fordjour E, Manful CF, Sey AA, Cannabis: a multifaceted plant with endless potentials. Front Pharmacol 2023;14. 10.3389/fphar.2023.1200269. PMC10308385 37397476

[R24] Gonzalo-Consuegra C, Santos-García I, García-Toscano L, Involvement of CB1 and CB2 receptors in neuroprotective effects of cannabinoids in experimental TDP-43 related frontotemporal dementia using male mice. Biomedicine & Pharmacotherapy 2024;174:116473. 10.1016/j.biopha.2024.116473. 38522237

[R25] Helcman M, Šmejkal K. Biological activity of Cannabis compounds: a modern approach to the therapy of multiple diseases. Phytochem Rev 2022;21(2):429–470. 10.1007/s11101-021-09777-x.

[R26] Hryhorowicz S, Kaczmarek-Ryś M, Zielińska A, Endocannabinoid System as a Promising Therapeutic Target in Inflammatory Bowel Disease – A Systematic Review. Front Immunol 2021;12. 10.3389/fimmu.2021.790803. PMC8727741 35003109

[R27] Ibsen MS, Connor M, Glass M. Cannabinoid CB1 and CB2 Receptor Signaling and Bias. Cannabis and Cannabinoid Research 2017;2(1):48–60. 10.1089/can.2016.0037. 28861504 PMC5436336

[R28] Kim T, Song B, Cho KS, Therapeutic Potential of Volatile Terpenes and Terpenoids from Forests for Inflammatory Diseases. Int J Mol Sci 2020;21(6):2187. 10.3390/ijms21062187. 32235725 PMC7139849

[R29] Kim Y-A, Wuchty S, Przytycka TM. Identifying causal genes and dysregulated pathways in complex diseases. PLoS Comput Biol 2011;7(3):e1001095. 10.1371/journal.pcbi.1001095. 21390271 PMC3048384

[R30] Koltai H, Namdar D. Cannabis Phytomolecule “Entourage”: From Domestication to Medical Use. Trends Plant Sci 2020;25(10):976–984. 10.1016/j.tplants.2020.04.007. 32417167

[R31] LaVigne JE, Hecksel R, Keresztes A, Cannabis sativa terpenes are cannabimimetic and selectively enhance cannabinoid activity. Sci Rep 2021;11(1):8232. 10.1038/s41598-021-87740-8. 33859287 PMC8050080

[R32] Li G, Peng X, Guo Y, Currently Available Strategies for Target Identification of Bioactive Natural Products. Front Chem 2021;9:761609. 10.3389/fchem.2021.761609. 34660543 PMC8515416

[R33] Li H-L. An Archaeological and Historical Account of Cannabis in China. Economic Botany 1974;28(4):437–448.

[R34] Liu Y, Chance MR. Pathway analyses and understanding disease associations. Curr Genet Med Rep 2013;1(4):10.1007/s40142-013-0025-3. 10.1007/s40142-013-0025-3. PMC3851310 24319650

[R35] Milacic M, Beavers D, Conley P, The Reactome Pathway Knowledgebase 2024. Nucleic Acids Research 2024;52(D1):D672–D678. 10.1093/nar/gkad1025. 37941124 PMC10767911

[R36] Namdar D, Anis O, Poulin P, Chronological Review and Rational and Future Prospects of Cannabis-Based Drug Development. Molecules 2020;25(20):4821. 10.3390/molecules25204821. 33092255 PMC7587964

[R37] Newman DJ, Cragg GM. Natural Products as Sources of New Drugs from 1981 to 2014. J Nat Prod 2016;79(3):629–661. 10.1021/acs.jnatprod.5b01055. 26852623

[R38] Ng T, Gupta V, Keshock MC. Tetrahydrocannabinol (THC). In: StatPearls StatPearls Publishing: Treasure Island (FL); 2024. 33085321

[R39] Nogueira MS, Koch O. The Development of Target-Specific Machine Learning Models as Scoring Functions for Docking-Based Target Prediction. J Chem Inf Model 2019;59(3):1238–1252. 10.1021/acs.jcim.8b00773. 30802041

[R40] Oláh A, Szekanecz Z, Bíró T. Targeting Cannabinoid Signaling in the Immune System: “High”-ly Exciting Questions, Possibilities, and Challenges. Front Immunol 2017;8. 10.3389/fimmu.2017.01487. PMC5686045 29176975

[R41] Pacher P, Bátkai S, Kunos G. The endocannabinoid system as an emerging target of pharmacotherapy. Pharmacol Rev 2006;58(3):389–462. 10.1124/pr.58.3.2. 16968947 PMC2241751

[R42] Raup-Konsavage WM, Carkaci-Salli N, Greenland K, Cannabidiol (CBD) Oil Does Not Display an Entourage Effect in Reducing Cancer Cell Viability in vitro. Med Cannabis Cannabinoids 2020;3(2):95–102. 10.1159/000510256. 34676344 PMC8489314

[R43] Raup-Konsavage WM, Sepulveda DE, Wang J, Antinociceptive Effects of Cannabichromene (CBC) in Mice: Insights from von Frey, Tail-Flick, Formalin, and Acetone Tests. Biomedicines 2023;12(1):83. 10.3390/biomedicines12010083. 38255191 PMC10813533

[R44] Ren G, Zhang X, Li Y, Large-scale whole-genome resequencing unravels the domestication history of Cannabis sativa. Sci Adv 2021;7(29):eabg2286. 10.1126/sciadv.abg2286. 34272249 PMC8284894

[R45] Scherma M, Muntoni AL, Riedel G, Cannabinoids and their therapeutic applications in mental disorders. Dialogues Clin Neurosci 2020;22(3):271–279. 10.31887/DCNS.2020.22.3/pfadda. 33162770 PMC7605020

[R46] Sepulveda DE, Vrana KE, Graziane NM, Combinations of Cannabidiol and Δ9-Tetrahydrocannabinol in Reducing Chemotherapeutic Induced Neuropathic Pain. Biomedicines 2022;10(10):2548. 10.3390/biomedicines10102548. 36289810 PMC9599350

[R47] Simiyu DC, Jang JH, Lee OR. Understanding Cannabis sativa L.: Current Status of Propagation, Use, Legalization, and Haploid-Inducer-Mediated Genetic Engineering. Plants (Basel) 2022;11(9):1236. 10.3390/plants11091236. 35567237 PMC9104644

[R48] The UniProt Consortium. UniProt: the Universal Protein Knowledgebase in 2023. Nucleic Acids Research 2023;51(D1):D523–D531. 10.1093/nar/gkac1052. 36408920 PMC9825514

[R49] Turcotte C, Blanchet M-R, Laviolette M, The CB2 receptor and its role as a regulator of inflammation. Cell Mol Life Sci 2016;73(23):4449–4470. 10.1007/s00018-016-2300-4. 27402121 PMC5075023

[R50] Varshney K, Patel A, Ansari S, Cannabinoids in Treating Parkinson’s Disease Symptoms: A Systematic Review of Clinical Studies. Cannabis and Cannabinoid Research 2023;8(5):716–730. 10.1089/can.2023.0023. 37253174

[R51] Wang J, Dokholyan NV. Yuel: Improving the Generalizability of Structure-free Compound-Protein Interaction Prediction. J Chem Inf Model 2022;62(3):463–471. 10.1021/acs.jcim.1c01531. 35103472 PMC9203246

[R52] Zeng X, Zhu S, Lu W, Target identification among known drugs by deep learning from heterogeneous networks. Chem Sci n.d.;11(7):1775–1797. 10.1039/c9sc04336e. PMC8150105 34123272

[R53] Zhang X, Wu F, Yang N, In silico Methods for Identification of Potential Therapeutic Targets. Interdiscip Sci Comput Life Sci 2022;14(2):285–310. 10.1007/s12539-021-00491-y. PMC8616973 34826045

[R54] Zhao X, Xia B, Cheng J, PKCε SUMOylation Is Required for Mediating the Nociceptive Signaling of Inflammatory Pain. Cell Reports 2020;33(1):108191. 10.1016/j.celrep.2020.108191. 33027667

[R55] Zhou L, Wang Y, Peng L, Identifying potential drug-target interactions based on ensemble deep learning. Front Aging Neurosci 2023;15:1176400. 10.3389/fnagi.2023.1176400. 37396659 PMC10309650

[R56] Zou S, Kumar U. Cannabinoid Receptors and the Endocannabinoid System: Signaling and Function in the Central Nervous System. Int J Mol Sci 2018;19(3):833. 10.3390/ijms19030833. 29533978 PMC5877694

